# Anti-SARS-CoV-2 Spike Protein RBD Antibody Levels After Receiving a Second Dose of ChAdOx1 nCov-19 (AZD1222) Vaccine in Healthcare Workers: Lack of Association With Age, Sex, Obesity, and Adverse Reactions

**DOI:** 10.3389/fimmu.2021.779212

**Published:** 2021-11-25

**Authors:** Sang Won Lee, Ji-Yong Moon, Sun-Kyung Lee, Hyun Lee, SeolHwa Moon, Sung Jun Chung, Yoomi Yeo, Tai Sun Park, Dong Won Park, Tae-Hyung Kim, Jang Won Sohn, Ho Joo Yoon, Sang-Heon Kim

**Affiliations:** ^1^ Department of Clinical Pharmacology and Therapeutics, Hanyang University Hospital, Seoul, South Korea; ^2^ Division of Pulmonary Medicine and Allergy, Department of Internal Medicine, Hanyang University College of Medicine, Seoul, South Korea; ^3^ Department of Mathematics, College of Natural Sciences, Hanyang University, Seoul, South Korea; ^4^ College of Nursing, Hanyang University, Seoul, South Korea

**Keywords:** COVID-19, vaccine, antibody, adverse reaction, age, sex, obesity

## Abstract

Response to vaccines generally varies according to individual factors of the vaccinated subjects such as demographics and immune status. While there are various reports of factors associated with immunogenicity of mRNA COVID-19 vaccines, little is known about those of adenovirus vector vaccines. We conducted a prospective observational study to assess the relationships of antibody level with age, sex, body mass index (BMI), and adverse reactions (ARs) to an adenovirus vector vaccine, ChAdOx1 nCoV-19. Healthcare workers who planned to receive both the first and second injections of the ChAdOx1 nCoV-19 vaccine at Hanyang University Hospital, Seoul, Korea, were enrolled in the study. Seven days after each injection, participants were asked to complete an online adverse reaction survey. In addition, anti-SARS-CoV-2 spike (S) protein receptor binding domain (RBD) antibody concentration was measured 4 weeks after the second injection. All participants (n = 447, 100%) showed serologic positivity (≥ 0.8 U/mL) 4 weeks after the second injection of ChAdOx1 nCoV-19 vaccine. Furthermore, the anti-SARS-CoV-2 S protein RBD concentration was similar among groups when stratified by age, sex, BMI, or presence and severity of AR; multivariable linear regression found no associations between antibody response to the ChAdOx1 nCoV-19 vaccine and age, BMI, sex, and vaccine-induced ARs. In conclusion, age, sex, obesity, and ARs were not associated with antibody responses after two doses of ChAdOx1 nCoV-19 vaccination.

## Introduction

Coronavirus disease 2019 (COVID-19) is caused by severe acute respiratory syndrome coronavirus 2 (SARS-CoV-2) and has spread worldwide to become the most serious health problem. To protect people from COVID-19 and provide protective immunity, various types of vaccines have been developed and administered to the public (1). While clinical trials and real-world studies showed the immunogenicity and efficacy of these vaccines at the population level (2–5), there is individual variation in immune responses to the vaccination, including antibody responses (6, 7). Experience with other vaccines has demonstrated a wide range of variability in response according to demographics and immune status factors of the vaccinated subjects (8).

While it is difficult to assess the immunogenicity of vaccines, measuring antibody level to SARS-CoV-2 in vaccinated subjects is accepted as a diagnostic test to determine vaccine efficacy despite caveats in interpretation of the results (9). Regarding mRNA COVID-19 vaccines (BNT162b2 and mRNA-1273 COVID-19 vaccines), various factors have been reported to be associated with low antibody responses, including old age (10–22), male sex (11, 13–15, 20, 22), higher body mass index (BMI) (11, 19, 20), medications, and comorbidities (23, 24). Moreover, adverse reactions (ARs) to vaccines are suggested to be related to higher antibody level (14, 15, 17). Contrarily, less is known about factors affecting antibody responses to adenovirus vector vaccines, such as the ChAdOx1 nCoV-19 (AZD1222) vaccine. In this prospective observational study, we measured the serum level of the anti-SARS-CoV-2 spike (S) protein receptor binding domain (RBD) antibody in healthcare workers who were vaccinated twice with the ChAdOx1 nCoV-19 vaccine and assessed the relationships of antibody level with age, sex, BMI, and ARs to the vaccine.

## Materials and Methods

### Study Population and Design

Eligible participants were healthcare workers (HCW) who received both the first (prime) and the second (booster) injections of ChAdOx1 nCoV-19 vaccine at Hanyang University Hospital, Seoul, Korea. The first and second injections were administered between March 8, 2021, and May 28, 2021, approximately 12 weeks apart. In a previous clinical study of ChAdOx1 nCoV-19 vaccine, the severity and intensity of local and systemic reactions was highest one day after vaccination (25). Therefore, seven days after each injection, participants were asked to complete an online AR survey to capture the AR profile within one week of receiving each vaccination. Serum samples were collected 4 weeks after the second injection of ChAdOx1 nCoV-19 vaccine for quantitative measurement of anti-SARS-CoV-2 spike S protein RBD antibody concentration. Written informed consent was obtained from each participant before any study-related procedure was performed.

### Adverse Event Assessment

The online AR survey was completed by all participants seven days after each injection of ChAdOx1 nCoV-19 vaccine. Demographic and solicited AR data were collected through a series of questionnaires. Demographic data included age, sex, height, weight, occupation, history of allergies, history of COVID-19 infection, comorbidities, and medication history. BMI was categorized into four groups (< 18.5, 18.5-22.9, 23.0-24.9, and ≥25.0 kg/m^2^) according to the Asian-Pacific definition of obesity (26). Based on the US Food and Drug Administration guidelines (27), the severity of solicited ARs was graded from 1 to 4 (grade 1, mild; grade 2, moderate; grade 3, severe; grade 4, potentially life threatening). Each AR was classified as systemic or local. The severity of ARs was based on the highest grade of solicited systemic or local AR during either injection.

### Quantification of Anti-SARS-CoV-2 Spike Protein Receptor Binding Domain Antibody

Quantification of anti-SARS-CoV-2 S protein RBD antibodies (including IgG) was performed using the Elecsys^®^ Anti-SARS-CoV-2 S immunoassay (Roche Diagnostics International Ltd, Rotkreuz, Switzerland). For each participant, a serum sample was collected 4 weeks after the second dose of ChAdOx1 nCoV-19 vaccine using tubes containing separating gel and was stored at approximately 4°C. The cutoff value for a positive result was defined as ≥ 0.80 U/mL (values < 0.80 U/mL were considered negative results) (28). The lower limit of quantitation was 0.40 U/mL, and samples with a concentration above 250 U/mL were diluted 10-fold for analysis (dilution was considered when calculating sample concentration). The measurement range was 0.40-2,500 U/mL, and values above the range were recorded as 2,500 U/mL.

### Statistical Analysis

Demographic characteristics were presented as number of subjects (%). We used Beeswarm Boxplot to show the distribution of the anti-SARS-CoV2 S protein RBD antibody concentration according to age, sex, BMI, and severity of ARs. The antibody titers were natural log (ln)-transformed as an outcome measure to ensure normality. Univariable and multivariable linear regression analyses were performed to verify the associations between immunogenicity and age, sex, BMI, and severity of ARs. To assess the independent relationship between immunogenicity and severity of ARs, we determined standardized beta coefficients (β) before and after adjusting for age, sex, and BMI. All statistical analyses were performed using SAS^®^ 9.4 (SAS Institute, Cary, NC, USA), and a two-sided *p*-value < 0.05 was considered statistically significant.

## Results

### Characteristics of the Subjects

A total of 447 HCWs who received both the first and second injections of ChAdOx1 nCoV-19 vaccine was enrolled in the study, and 388 (86.8%) of them were female (**Table 1**). Age of the subjects ranged from 21 to 63 years, with a mean ± standard deviation (SD) of 40.6 ± 10.9 years. The mean BMI was 22.4 ± 3.1 kg/m^2^, and 19.7% (n = 88) of subjects were obese (BMI ≥ 25.0 kg/m^2^). The majority of participants was nurses or nursing assistants (n = 282, 63.1%), followed by laboratory staff or physical therapists (n = 48, 10.7%) and physicians (n = 26, 5.8%). Most of the participants did not have comorbidities, though some subjects had hypertension (3.1%), allergy (1.8%), diabetes mellitus (1.8%), asthma (0.7%), chronic liver disease (0.7%), or chronic heart disease (0.5%). In addition, one participant (0.2%) reported previous infection of COVID-19.

**Table 1:** Characteristics of the study population.

	Subjects (n = 447)
**Age (years)**	
21-30	120 (26.9)
31-40	80 (17.9)
41-50	152 (34.0)
51-63	95 (21.2)
**Sex**	
Female	388 (86.8)
Male	59 (13.2)
**Body mass index (kg/m^2^)**	
< 18.5	32 (7.2)
18.5-22.9	251 (56.1)
23.0-24.9	76 (17.0)
≥25.0	88 (19.7)
**Occupation**	
Nurse/nursing assistant	282 (63.1)
Physician	26 (5.8)
Laboratory staff or physical therapist	48 (10.7)
Other	91 (20.4)
**Comorbidities**	
Hypertension	14 (3.1)
Any allergy other than asthma	8 (1.8)
Diabetes mellitus	8 (1.8)
Asthma	3 (0.7)
Chronic liver disease	3 (0.7)
Chronic heart disease	2 (0.5)
Adverse reactions to any vaccination*	
None	13 (2.9)
Grade 1	46 (10.3)
Grade 2	182 (40.7)
Grade 3 or 4	206 (46.1)

Values are presented as number (%) of subjects.

^*^Severity of adverse reactions was determined as the highest grade of local or systemic adverse reaction to any ChAdOx1 nCoV-19 vaccination.

### Adverse Reactions to Vaccine

Most participants (n = 434, 97.1%) experienced at least one AR (any local or systemic AR) during days 0-7 after either injection (first or second). Overall, the most frequently reported local AR was pain (n = 406, 91.8%), followed by tenderness (n = 386, 86.3%) and induration (n = 138, 30.9%), while the most frequently reported systemic AR was fatigue (n = 391, 87.5%), followed by muscle pain (n = 367, 82.1%), chills (n = 309, 69.1%), and headache (n = 309, 69.1%). In general, both systemic (first injection, n = 389, 87%; second injection, n = 196, 43.8%) and local (first injection, n = 386, 86.4%; second injection, n = 219, 49%) ARs were less frequent after the second injection. In terms of severity of ARs, nearly half of the participants (n = 206, 46.1%) showed at least one grade 3 or 4 AR. At 0-7 days following either (first or second) injection, the most frequently reported grade 3 to grade 4 local AR was tenderness (26.8%), followed by pain (5.8%) and itching (4.4%), while the most frequently reported grade 3 to grade 4 local AR was chills (18.6%), followed by fatigue (18.2%), muscle pain (17%), and fever (6.1%) (**Supplementary Figure 1**).

### Associations Between Immunogenicity and Age, Sex, BMI, and Severity of AR

**Figure 1:**
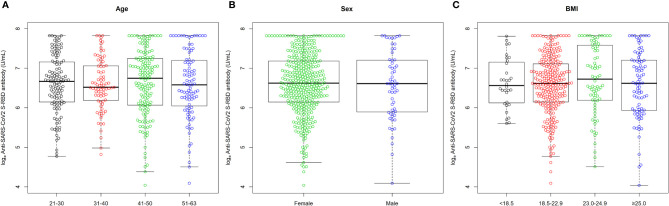
Distribution of log-transformed (ln) antibody concentration at 4 weeks after the second injection of ChAdOx1 nCoV-19 vaccine according to **(A)** age (years) group, **(B)** sex, and **(C)** body mass index (kg/m^2^) group. Antibody was expressed as the natural logarithm of concentration of anti-SARS-CoV-2 S protein RBD. Upper and lower whiskers represent the highest and lowest data points, respectively, excluding any outliers. The horizontal lines in the middle, top, and bottom of the box represent the median, 75^th^ percentile, and 25^th^ percentile, respectively.

**Figure 2:**
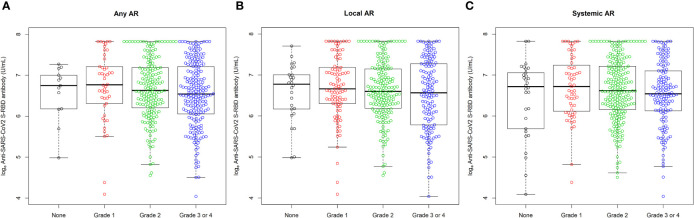
Distribution of log-transformed (ln) antibody concentration at 4 weeks after the second injection of ChAdOx1 nCoV-19 vaccine according to severity of **(A)** any adverse reactions, **(B)** local adverse reactions, and **(C)** systemic adverse reactions to any ChAdOx1 nCoV-19 vaccination. Antibody was expressed as the natural logarithm of concentrations of anti-SARS-CoV-2 S protein RBD. Upper and lower whiskers represent the highest and lowest data points, respectively, excluding any outliers. The horizontal lines in the middle, top, and bottom of the box represent the median, 75^th^ percentile, and 25^th^ percentile, respectively.

All participants (n = 447, 100%) showed serologic positivity (≥ 0.8 U/mL) 4 weeks after the second injection of ChAdOx1 nCoV-19 vaccine. Among them, the median (IQR) anti-SARS-CoV-2 S protein RBD concentration was 747.0 (455.0-1324.0) U/mL. The anti-SARS-CoV-2 S protein RBD concentrations were similar among groups when stratified by age (median for 21-30 years old = 787.0 U/mL; median for 31-40 years old = 678.0 U/mL; median for 41-50 years old = 852.0 U/mL; median for 51-63 years old = 720.0 U/mL), sex (median for female = 753.0 U/mL; median for male = 744.0 U/mL), or BMI (median for <18.5 kg/m^2^ = 707.5 U/mL; median for 18.5-22.9 kg/m^2^ = 747.0 U/mL; median for 23.0-24.9 kg/m^2^ = 832.5 U/mL; median for ≥25.0 kg/m^2^ = 748.5 U/mL) (**Figure 1**). The concentration also was similar when stratified by presence and severity of both local and systemic ARs (median for no local AR = 877.5 U/mL; median for Grade 1 local AR = 781.5 U/mL; median for Grade 2 local AR = 734.0 U/mL; median for Grade 3 or 4 local AR = 709.0 U/mL; median for no systemic ARs = 828.0 U/mL; median for Grade 1 systemic ARs = 830.0 U/mL; median for Grade 2 systemic AR = 748.5 U/mL; median for Grade 3 or 4 systemic AR = 693.0 U/mL) (**Figure 2**). Furthermore, a multivariable linear regression analysis of anti-SARS-CoV-2 S protein RBD concentration found no significant association of antibody concentration with age, sex, BMI, or severity of AR (**Table 2**).

**Table 2:** Linear regression models for natural log-transformed anti-SARS-CoV2 S-RBD antibody at 4 weeks after the second injection of ChAdOx1 nCoV-19 vaccine.

	Univariable analyses		Multivariable analyses					
	Standardized β	p-value	Standardized β^a^	p-value	Standardized β^b^	p-value	Standardized β^c^	p-value
**Age (years)**	-0.037	0.4327	-0.071	0.1721	-0.059	0.2465	-0.056	0.2899
**Female**	0.021	0.6613	0.039	0.4271	0.038	0.4442	0.029	0.5550
**BMI (kg/m^2^)**	0.010	0.8375	0.038	0.4649	0.037	0.4716	0.032	0.5370
**Severity of any AR**	-0.072	0.1266	-0.095	0.0563	–		–	
**Severity of local AR**	-0.063	0.1817	–		-0.079	0.1079	–	
**Severity of systemic AR**	-0.017	0.7132	–		–		-0.035	0.4861

AR, adverse reaction; BMI, body mass index.

Multivariable models adjusted for age, sex, BMI, and ^a^ any AR; ^b^ local AR; ^c^ systemic AR.

## Discussion

The efficacy and immunogenicity of COVID-19 vaccines vary among recipients. In this prospective observational study, we determined antibody levels 4 weeks after the second dose of the ChAdOx1 nCoV-19 vaccine (AZD1222) in HCWs and explored the relationships with host factors including age, sex, obesity, and vaccine-induced adverse reactions. We found a lack of association between antibody response to the ChAdOx1 nCoV-19 vaccine and age, sex, obesity, and vaccine-induced ARs.

Protective immunity of the COVID-19 vaccines has been observed in T cell and B cell responses (7, 29). Following immunization, the dendritic cells detect vaccine antigens and activate T cells in draining lymph nodes, leading to B cell proliferation and production of antibodies specific for the vaccine proteins (30). Heterogeneity in immune responses among vaccine recipients can explain the variation in COVID-19 vaccine efficacy. It is well known that host-related factors such as age, sex, and obesity can impact vaccine efficacy (31). Recent studies on the immune responses of mRNA COVID-19 vaccines reported lower antibody responses in subjects who are of older age, male, and obese (32). Contrary to the large body of evidence regarding this relationship in mRNA COVID-19 vaccines, such as the BNT162b2 COVID-19 vaccine, little is known about the impact of host factors on antibody responses to viral vector vaccines.

Older patients with COVID-19 showed decreased adaptive immune responses (33), which might contribute to higher mortality and worse outcomes than those in younger patients. In elderly people, decreased immune responses to other vaccines have been observed and explained by immunosenescence (34, 35). For immunogenicity of the BNT162b2 COVID-19 vaccine, a lower antibody response in older adults (65-85 years) than in younger adults (18–55) was observed in the clinical trial (36). These findings were confirmed from global real-world studies regardless of race (10–22). Interestingly, antibody responses to the mRNA-1273 COVID-19 vaccine in older adults were similar to those of younger adults in a clinical trial (37). While immune responses, including antibody responses, to the ChAdOx1 nCoV-19 vaccine were similar between young and old subjects in clinical trials (7, 38), real-world evidence is needed to evaluate the possible relationship between age and antibody responses of the ChAdOx1 nCoV-19 vaccine. Consistent with the observations from clinical trials, we found that age is not associated with antibody responses to the ChAdOx1 nCoV-19 vaccine.

In addition to age, associations of sex with vaccine responses have been acknowledged (39, 40). With the exception of a few vaccines, males are more likely to have lower antibody responses than females. Given the higher mortality and poorer outcomes of COVID-19 in males (41), it is very important to assess the impact of sex on the immunogenicity of COVID-19 vaccines. Previous studies on the association between sex and antibody responses to the BNT162b2 COVID-19 vaccine have not yielded consistent results. While some studies reported lower antibody concentration in males than in females (11, 13–15, 20, 22), this association was not observed in other studies (10, 12, 18, 42). In the present study, we found no significant difference in antibody response to the ChAdOx1 nCoV-19 vaccine between males and females. Our findings confirm previous observations from clinical trials that sex is not associated with cellular or antibody responses to the ChAdOx1 nCoV-19 vaccine (7).

Obesity, or high BMI, is a risk factor for morbidity and mortality of COVID-19 (43). Since obesity is known to interfere with the immune response to some vaccines, such as the hepatitis B and A vaccines and the influenza vaccine (44), concerns arose regarding possibly low efficacy and immunogenicity to COVID-19 vaccines in obese people (45). Clinical trials of mRNA COVID-19 vaccines (BNT162b2 and mRNA-1273 COVID-19 vaccines) showed similar efficacy between people with or without obesity (2, 3). However, Pellini and colleagues reported decreased antibody responses to the first dose of BNT162b2 COVID-19 vaccine in a obesity or pre-obesity group among HCWs in Italy (19). In their analysis after the booster dose of the BNT162b2 COVID-19 vaccine, BMI was not significantly associated with antibody response (20). In the present study, we observed no difference in the concentration of anti-SARS-CoV-2 S protein RBD antibody between BMI groups in HCWs vaccinated with prime and booster doses of the ChAdOx1 nCoV-19 vaccine. These findings suggest that obesity is not related with antibody responses to the ChAdOx1 nCoV-19 vaccine.

The reasons are uncertain for why age and sex did not show association with antibody response after ChAdOx1 nCoV-19 vaccine in this study while old age and male sex are reported to be associated with low antibody response after mRNA COVID-19 vaccines. However, neutralizing antibody levels were shown to be relatively higher in mRNA COVID-19 vaccines, while ChAdOx1 nCoV-19 vaccine was shown to induce polyfunctional antibodies, which are capable of mediating neutralization and other antibody-dependent effector mechanisms (46). Therefore, the differences regarding the association of age and sex with antibody response may be caused by the fact that mRNA COVID-19 and ChAdOx1 nCoV-19 vaccines induce different kinds of antibodies.

Adverse reactions to vaccines can indicate a strong immune response (47). There are conflicting results on the associations between reactogenicity to the COVID-19 vaccine and antibody responses. Kontou et al. found that HCWs with any AR to the first or second injection of the BNT162b2 COVID-19 vaccine had higher antibody titers than those without any AR (14). Contrarily, other studies reported that the severity of local or systemic adverse reactions to the BNT162b2 COVID-19 vaccine is not related to antibody reactions (48, 49). Regarding the ChAdOx1 nCoV-19 vaccine, a previous study showed an association between antibody response and systemic AR, but not local reactions, after the first dose (50). In our study, we examined whether ARs to the first and second doses of the ChAdOx1 nCoV-19 vaccine were associated with anti-SARS-CoV-2 S protein RBD antibody titers 4 weeks after the second dose. Contrary to previous reports, we did not find significant difference in antibody concentration according to presence or severity of local or systemic adverse reactions. Furthermore, in the analysis of the ARs after the first and second vaccinations, there were no significant associations (**Supplementary Figures 2**, **3**). The inconsistent results can be explained by the difference in time points of the antibody measurements (after a single dose versus after two doses) and statistical analysis methods (univariable versus multivariable) among studies. Given that ARs occur more frequently in females and younger people (51), the associations between adverse reactions and antibody reactions should be analyzed after adjusting for these possible confounding factors.

The major strength of this study is that we demonstrated that subjects who were vaccinated with two doses of the ChAdOx1 nCoV-19 vaccine had acceptable antibody responses, and host factors of age, sex, BMI, and ARs were not associated with antibody reactions in a prospective observational real-world study. However, this study has limitations that should be acknowledged. First, while we measured anti-SARS-CoV-2 antibody, not the neutralizing antibody or cellular immune response, it cannot project overall immune response and neutralizing activity. Given the strong correlation between anti-SARS-CoV-2 antibody and neutralizing antibody concentrations (29), we assume that anti-SARS-CoV-2 antibody reflects protective immunity. Second, because antibody level can vary over time, a single measurement does not reflect overall antibody response and immunogenicity of the recipients. Therefore, we selected the day of antibody measurement based on knowledge that maximum antibody titer was observed 28 days after vaccination according to a previous clinical trial (7). Third, since this was a single-center study and the subjects were HCWs, the study population may not properly represent the general population. Older adults were not enrolled and majority of the enrolled subjects (86.8%) were females. Thus, we could not determine the impact of older age (over 65 years) on antibody responses, and our claim that sex has little impact on antibody response is somewhat limited. While previous clinical trials in a small number of subjects showed similar immunogenicity among age groups (38) and sexes (49), further studies are warranted in a real-world setting with a large number of subjects. Fourth, there is a possibility that other host factors such as comorbidities or medications affected antibody responses to COVID vaccines. Since the subjects of this study were HCWs who had relatively fewer comorbidities than the normal population, we could not address this issue in this study.

In conclusion, subjects showed fair antibody responses after two doses of the ChAdOx1 nCoV-19 vaccine. Age, sex, and obesity were not associated with antibody responses after vaccinations. In addition, ARs to the vaccines did not predict the antibody responses to the ChAdOx1 nCoV-19 vaccine. These findings help assure the public and vaccine recipients of vaccine efficacy and immunogenicity.

## Data Availability Statement

The raw data supporting the conclusions of this article will be made available by the authors, without undue reservation.

## Ethics Statement

The studies involving human participants were reviewed and approved by Institutional Review Board of Hanyang University Seoul Hospital. The patients/participants provided their written informed consent to participate in this study.

## Author Contributions

SL and J-YM contributed equally to data analysis, data interpretation, and writing of the manuscript. S-KL, HL, and SL contributed to statistical analyses. S-HK and SL designed the study. YY, TP, DP, and T-HK contributed to data acquisition and clearing. JS, HY, and S-HK oversaw data analysis, data interpretation, and writing of the manuscript. All authors contributed to the article and approved the submitted version.

## Funding

This research was supported by a grant from the Korea Health Technology R&D Project through the Korea Health Industry Development Institute (KHIDI), funded by the Ministry of Health & Welfare, Republic of Korea (grant number: HI19C0218), and by the Bio & Medical Technology Development Program of the National Research Foundation (NRF) funded by the Korean government (MSIT) (No. 2019M3E5D1A01069363).

## Conflict of Interest

The authors declare that the research was conducted in the absence of any commercial or financial relationships that could be construed as a potential conflict of interest.

## Publisher’s Note

All claims expressed in this article are solely those of the authors and do not necessarily represent those of their affiliated organizations, or those of the publisher, the editors and the reviewers. Any product that may be evaluated in this article, or claim that may be made by its manufacturer, is not guaranteed or endorsed by the publisher.

## References

[1] Callaway E. The Race for Coronavirus Vaccines: A Graphical Guide. Nature (2020) 580(7805):576–7. doi: 10.1038/d41586-020-01221-y 32346146

[2] Polack FP Thomas SJ Kitchin N Absalon J Gurtman A Lockhart S. Safety and Efficacy of the BNT162b2 mRNA Covid-19 Vaccine. N Engl J Med (2020) 383(27):2603–15. doi: 10.1056/NEJMoa2034577 PMC7745181 33301246

[3] Baden LR El Sahly HM Essink B Kotloff K Frey S Novak R. Efficacy and Safety of the mRNA-1273 SARS-CoV-2 Vaccine. N Engl J Med (2021) 384(5):403–16. doi: 10.1056/NEJMoa2035389 PMC7787219 33378609

[4] Haas EJ Angulo FJ McLaughlin JM Anis E Singer SR Khan F. Impact and Effectiveness of mRNA BNT162b2 Vaccine Against SARS-CoV-2 Infections and COVID-19 Cases, Hospitalisations, and Deaths Following a Nationwide Vaccination Campaign in Israel: An Observational Study Using National Surveillance Data. Lancet (2021) 397(10287):1819–29. doi: 10.1016/s0140-6736(21)00947-8 PMC8099315 33964222

[5] Voysey M Clemens SAC Madhi SA Weckx LY Folegatti PM Aley PK. Safety and Efficacy of the ChAdOx1 Ncov-19 Vaccine (AZD1222) Against SARS-CoV-2: An Interim Analysis of Four Randomised Controlled Trials in Brazil, South Africa, and the UK. Lancet (2021) 397(10269):99–111. doi: 10.1016/s0140-6736(20)32661-1 33306989 PMC7723445

[6] Bradley T Grundberg E Selvarangan R LeMaster C Fraley E Banerjee D. Antibody Responses After a Single Dose of SARS-CoV-2 mRNA Vaccine. N Engl J Med (2021) 384(20):1959–61. doi: 10.1056/NEJMc2102051 PMC8008753 33755375

[7] Ewer KJ Barrett JR Belij-Rammerstorfer S Sharpe H Makinson R Morter R. T Cell and Antibody Responses Induced by a Single Dose of ChAdOx1 Ncov-19 (AZD1222) Vaccine in a Phase 1/2 Clinical Trial. Nat Med (2021) 27(2):270–8. doi: 10.1038/s41591-020-01194-5 33335323

[8] Shen-Orr SS. And Furman, D. Variability in the Immune System: Of Vaccine Responses and Immune States. Curr Opin Immunol (2013) 25(4):542–7. doi: 10.1016/j.coi.2013.07.009 PMC3788704 23953808

[9] Hodgson SH Mansatta K Mallett G Harris V Emary KRW Pollard AJ. And What Defines an Efficacious COVID-19 Vaccine? A Review of the Challenges Assessing the Clinical Efficacy of Vaccines Against SARS-CoV-2. Lancet Infect Dis (2021) 21(2):e26–35. doi: 10.1016/s1473-3099(20)30773-8 PMC7837315 33125914

[10] Abu Jabal K Ben-Amram H Beiruti K Batheesh Y Sussan C Zarka S. Impact of Age, Ethnicity, Sex and Prior Infection Status on Immunogenicity Following a Single Dose of the BNT162b2 mRNA COVID-19 Vaccine: Real-World Evidence From Healthcare Workers, Israel, December 2020 to January 2021. Euro Surveill (2021) 26(6). doi: 10.2807/1560-7917.Es.2021.26.6.2100096 PMC7879501 33573712

[11] Bayart JL Morimont L Closset M Wieërs G Roy T Gerin V. Confounding Factors Influencing the Kinetics and Magnitude of Serological Response Following Administration of BNT162b2. Microorganisms (2021) 9(6). doi: 10.3390/microorganisms9061340 PMC8235462 34205564

[12] Boyarsky BJ Werbel WA Avery RK Tobian AAR Massie AB Segev DL. Immunogenicity of a Single Dose of SARS-CoV-2 Messenger RNA Vaccine in Solid Organ Transplant Recipients. Jama (2021) 325(17):1784–6. doi: 10.1001/jama.2021.4385 PMC7961463 33720292

[13] Kageyama T Ikeda K Tanaka S Taniguchi T Igari H Onouchi Y. Antibody Responses to BNT162b2 mRNA COVID-19 Vaccine and Their Predictors Among Healthcare Workers in a Tertiary Referral Hospital in Japan. Clin Microbiol Infect (2021). doi: 10.1016/j.cmi.2021.07.042 PMC8349446 34375755

[14] Kontou E Ranellou K Zoulas D Bletsa A Rompola E Piperaki ET. Antibody Response Following a Two-Dose mRNA Vaccination Regimen, in Health Care Workers of a Tertiary Hospital in Athens, Greece. J Pers Med (2021) 11(6):576. doi: 10.3390/jpm11060576 34205301 PMC8234272

[15] Lo Sasso B Giglio RV Vidali M Scazzone C Bivona G Gambino CM. Evaluation of Anti-SARS-Cov-2 S-RBD IgG Antibodies After COVID-19 mRNA BNT162b2 Vaccine. Diagnostics (Basel) (2021) 11(7):1135. doi: 10.3390/diagnostics11071135 34206567 PMC8306884

[16] Müller L Andrée M Moskorz W Drexler I Walotka L Grothmann R. Age-Dependent Immune Response to the Biontech/Pfizer BNT162b2 COVID-19 Vaccination. Clin Infect Dis (2021). doi: 10.1093/cid/ciab381 PMC8135422 33906236

[17] Naaber P Jürjenson V Adamson A Sepp E Tserel L Kisand K. Antibody Response After COVID-19 mRNA Vaccination in Relation to Age, Sex, and Side Effects. medRxiv (2021) 2021.04.19.21255714. doi: 10.1101/2021.04.19.21255714

[18] Padoan A Dall'Olmo L Rocca FD Barbaro F Cosma C Basso D. Antibody Response to First and Second Dose of BNT162b2 in a Cohort of Characterized Healthcare Workers. Clin Chim Acta (2021) 519:60–3. doi: 10.1016/j.cca.2021.04.006 PMC8056941 33857476

[19] Pellini R Venuti A Pimpinelli F Abril E Blandino G Campo F. Early Onset of SARS-COV-2 Antibodies After First Dose of BNT162b2: Correlation With Age, Gender and BMI. Vaccines (Basel) (2021) 9(7):685. doi: 10.3390/vaccines9070685 34206312 PMC8310011

[20] Pellini R Venuti A Pimpinelli F Abril E Blandino G Campo F. Initial Observations on Age, Gender, BMI and Hypertension in Antibody Responses to SARS-CoV-2 BNT162b2 Vaccine. EClinicalMedicine (2021) 36:100928. doi: 10.1016/j.eclinm.2021.100928 34109307 PMC8177433

[21] Salvagno GL Henry BM di Piazza G Pighi L De Nitto S Bragantini D. Anti-SARS-CoV-2 Receptor-Binding Domain Total Antibodies Response in Seropositive and Seronegative Healthcare Workers Undergoing COVID-19 mRNA BNT162b2 Vaccination. Diagnostics (Basel) (2021) 11(5):832. doi: 10.3390/diagnostics11050832 34064509 PMC8147939

[22] Terpos E Trougakos IP Apostolakou F Charitaki I Sklirou AD Mavrianou N. Age-Dependent and Gender-Dependent Antibody Responses Against SARS-CoV-2 in Health Workers and Octogenarians After Vaccination With the BNT162b2 mRNA Vaccine. Am J Hematol (2021) 96(7):E257–9. doi: 10.1002/ajh.26185 PMC8250071 33837984

[23] Deepak P Kim W Paley MA Yang M Carvidi AB El-Qunni AA. Glucocorticoids and B Cell Depleting Agents Substantially Impair Immunogenicity of mRNA Vaccines to SARS-CoV-2. medRxiv (2021). doi: 10.1101/2021.04.05.21254656

[24] Grupper A Sharon N Finn T Cohen R Israel M Agbaria A. Humoral Response to the Pfizer BNT162b2 Vaccine in Patients Undergoing Maintenance Hemodialysis. Clin J Am Soc Nephrol (2021) 16(7):1037–42. doi: 10.2215/cjn.03500321 PMC8425628 33824157

[25] Folegatti PM Ewer KJ Aley PK Angus B Becker S Belij-Rammerstorfer S. Safety and Immunogenicity of the ChAdOx1 Ncov-19 Vaccine Against SARS-CoV-2: A Preliminary Report of a Phase 1/2, Single-Blind, Randomised Controlled Trial. Lancet (2020) 396(10249):467–78. doi: 10.1016/S0140-6736(20)31604-4 PMC7445431 32702298

[26] Kim MK Lee W-Y Kang J-H Kang J-H Kim BT Kim SM. Clinical Practice Guidelines for Overweight and Obesity in Korea. Endocrinol Metab (2014) 29(4):405–9. doi: 10.3803/EnM.2014.29.4.405 PMC4285036 25559568

[27] Center for Biologics Evaluation and Research, Food and Drug Administration. Guidance for Industry, Toxicity Grading Scale for Healthy Adult and Adolescent Volunteers Enrolled in Preventive Vaccine Clinical Trials. U.S.: FDA. Available at https://www.fda.gov/media/73679/download (Accessed 16th April 2021).

[28] Riester E Findeisen P Hegel JK Kabesch M Ambrosch A Rank CM. Performance Evaluation of the Roche Elecsys Anti-SARS-CoV-2 S Immunoassay. MedRxiv (2021). doi: 10.1101/2021.03.02.21252203 PMC8393518 34461153

[29] Sahin U Muik A Vogler I Derhovanessian E Kranz LM Vormehr M. BNT162b2 Vaccine Induces Neutralizing Antibodies and Poly-Specific T Cells in Humans. Nature (2021) 595(7868):572–7. doi: 10.1038/s41586-021-03653-6 34044428

[30] Pollard AJ Bijker EM. A Guide to Vaccinology: From Basic Principles to New Developments. Nat Rev Immunol (2021) 21(2):83–100. doi: 10.1038/s41577-020-00479-7 33353987 PMC7754704

[31] Dhakal S Klein SL. Host Factors Impact Vaccine Efficacy: Implications for Seasonal and Universal Influenza Vaccine Programs. J Virol (2019) 93(21):e00797–19. doi: 10.1128/jvi.00797-19 PMC6803252 31391269

[32] Lippi G Henry BM Plebani M. Anti-SARS-CoV-2 Antibodies Testing in Recipients of COVID-19 Vaccination: Why, When, and How? Diagnostics (Basel) (2021) 11(6):941. doi: 10.3390/diagnostics11060941 34070341 PMC8228868

[33] Rydyznski Moderbacher C Ramirez SI Dan JM Grifoni A Hastie KM Weiskopf D. Antigen-Specific Adaptive Immunity to SARS-CoV-2 in Acute COVID-19 and Associations With Age and Disease Severity. Cell (2020) 183(4):996–1012.e19. doi: 10.1016/j.cell.2020.09.038 33010815 PMC7494270

[34] Lang PO Govind S Bokum AT Kenny N Matas E Pitts D. Immune Senescence and Vaccination in the Elderly. Curr Top Med Chem (2013) 13(20):2541–50. doi: 10.2174/15680266113136660181 24066892

[35] Crooke SN Ovsyannikova IG Poland GA Kennedy RB. Immunosenescence and Human Vaccine Immune Responses. Immun Ageing (2019) 16:25. doi: 10.1186/s12979-019-0164-9 31528180 PMC6743147

[36] Walsh EE Frenck RW Jr. Falsey AR Kitchin N Absalon J Gurtman A. Safety and Immunogenicity of Two RNA-Based Covid-19 Vaccine Candidates. N Engl J Med (2020) 383(25):2439–50. doi: 10.1056/NEJMoa2027906 PMC7583697 33053279

[37] Anderson EJ Rouphael NG Widge AT Jackson LA Roberts PC Makhene M. Safety and Immunogenicity of SARS-CoV-2 mRNA-1273 Vaccine in Older Adults. N Engl J Med (2020) 383(25):2427–38. doi: 10.1056/NEJMoa2028436 PMC7556339 32991794

[38] Ramasamy MN Minassian AM Ewer KJ Flaxman AL Folegatti PM Owens DR. Safety and Immunogenicity of ChAdOx1 Ncov-19 Vaccine Administered in a Prime-Boost Regimen in Young and Old Adults (COV002): A Single-Blind, Randomised, Controlled, Phase 2/3 Trial. Lancet (2021) 396(10267):1979–93. doi: 10.1016/s0140-6736(20)32466-1 PMC7674972 33220855

[39] Fischinger S Boudreau CM Butler AL Streeck H Alter G. Sex Differences in Vaccine-Induced Humoral Immunity. Semin Immunopathol (2019) 41(2):239–49. doi: 10.1007/s00281-018-0726-5 PMC6373179 30547182

[40] Zimmermann P Curtis N. Factors That Influence the Immune Response to Vaccination. Clin Microbiol Rev (2019) 32(2):e00084–18. doi: 10.1128/cmr.00084-18 PMC6431125 30867162

[41] Peckham H de Gruijter NM Raine C Radziszewska A Ciurtin C Wedderburn LR. Male Sex Identified by Global COVID-19 Meta-Analysis as a Risk Factor for Death and ITU Admission. Nat Commun (2020) 11(1):6317. doi: 10.1038/s41467-020-19741-6 33298944 PMC7726563

[42] Levi R Azzolini E Pozzi C Ubaldi L Lagioia M Mantovani A. A Cautionary Note on Recall Vaccination in Ex-COVID-19 Subjects. medRxiv (2021) 2021.02.01.21250923. doi: 10.1101/2021.02.01.21250923

[43] Popkin BM Du S Green WD Beck MA Algaith T Herbst CH. Individuals With Obesity and COVID-19: A Global Perspective on the Epidemiology and Biological Relationships. Obes Rev (2020) 21(11):e13128. doi: 10.1111/obr.13128 32845580 PMC7461480

[44] Painter SD Ovsyannikova IG Poland GA. The Weight of Obesity on the Human Immune Response to Vaccination. Vaccine (2015) 33(36):4422–9. doi: 10.1016/j.vaccine.2015.06.101 PMC4547886 26163925

[45] Townsend MJ Kyle TK Stanford FC. COVID-19 Vaccination and Obesity: Optimism and Challenges. Obes (Silver Spring) (2021) 29(4):634–5. doi: 10.1002/oby.23131 PMC7990687 33506642

[46] Sadarangani M Marchant A Kollmann TR. Immunological Mechanisms of Vaccine-Induced Protection Against COVID-19 in Humans. Nat Rev Immunol (2021) 21(8):475–84. doi: 10.1038/s41577-021-00578-z PMC8246128 34211186

[47] Zhuang CL Lin ZJ Bi ZF Qiu LX Hu FF Liu XH. Inflammation-Related Adverse Reactions Following Vaccination Potentially Indicate a Stronger Immune Response. Emerg Microbes Infect (2021) 10(1):365–75. doi: 10.1080/22221751.2021.1891002 PMC7928063 33583360

[48] Coggins SAA Laing ED Olsen CH Goguet E Moser M Jackson-Thompson BM. Adverse Effects and Antibody Titers in Response to the BNT162b2 mRNA COVID-19 Vaccine in a Prospective Study of Healthcare Workers. medRxiv (2021). doi: 10.1101/2021.06.25.21259544 PMC8759445 35047649

[49] Hwang YH Song KH Choi Y Go S Choi SJ Jung J. Can Reactogenicity Predict Immunogenicity After COVID-19 Vaccination? Korean J Intern Med (2021) 36(6):1486–91. doi: 10.3904/kjim.2021.210 PMC8588964 34038996

[50] Park JY Choi SH Chung JW Hwang MH Kim MC. Systemic Adverse Events and Use of Antipyretics Predict the Neutralizing Antibody Positivity Early After the First Dose of ChAdOx1 Coronavirus Disease Vaccine. J Clin Med (2021) 10(13):2844. doi: 10.3390/jcm10132844 34199053 PMC8268750

[51] Bae S Lee YW Lim SY Lee JH Lim JS Lee S. Adverse Reactions Following the First Dose of ChAdOx1 Ncov-19 Vaccine and BNT162b2 Vaccine for Healthcare Workers in South Korea. J Korean Med Sci (2021) 36(17):e115. doi: 10.3346/jkms.2021.36.e115 33942579 PMC8093607

