# Supplementary material for: Anti-SARS-CoV-2 Spike Protein RBD Antibody Levels After Receiving a Second Dose of ChAdOx1 nCov-19 (AZD1222) Vaccine in Healthcare Workers: Lack of Association With Age, Sex, Obesity, and Adverse Reactions
Source: Front Immunol. 2021 Nov 25;12:779212. doi: 10.3389/fimmu.2021.779212 (PMC8654782; doi:10.3389/fimmu.2021.779212)
Supplement: Supplementary file 1 [file Image_1.pdf]

Supplementary Figure 1. Rates of local and systemic adverse reactions (ARs) 7 days after first and second injections of ChAdOx1 nCoV-19 vaccine. (A) local adverse reactions, (B) systemic adverse reactions. (1st, rate of AR 7 days after the first injection of ChAdOx1 nCoV-19 vaccine; 2nd, rate of AR 7 days after the second injection of ChAdOx1 nCoV-19 vaccine)

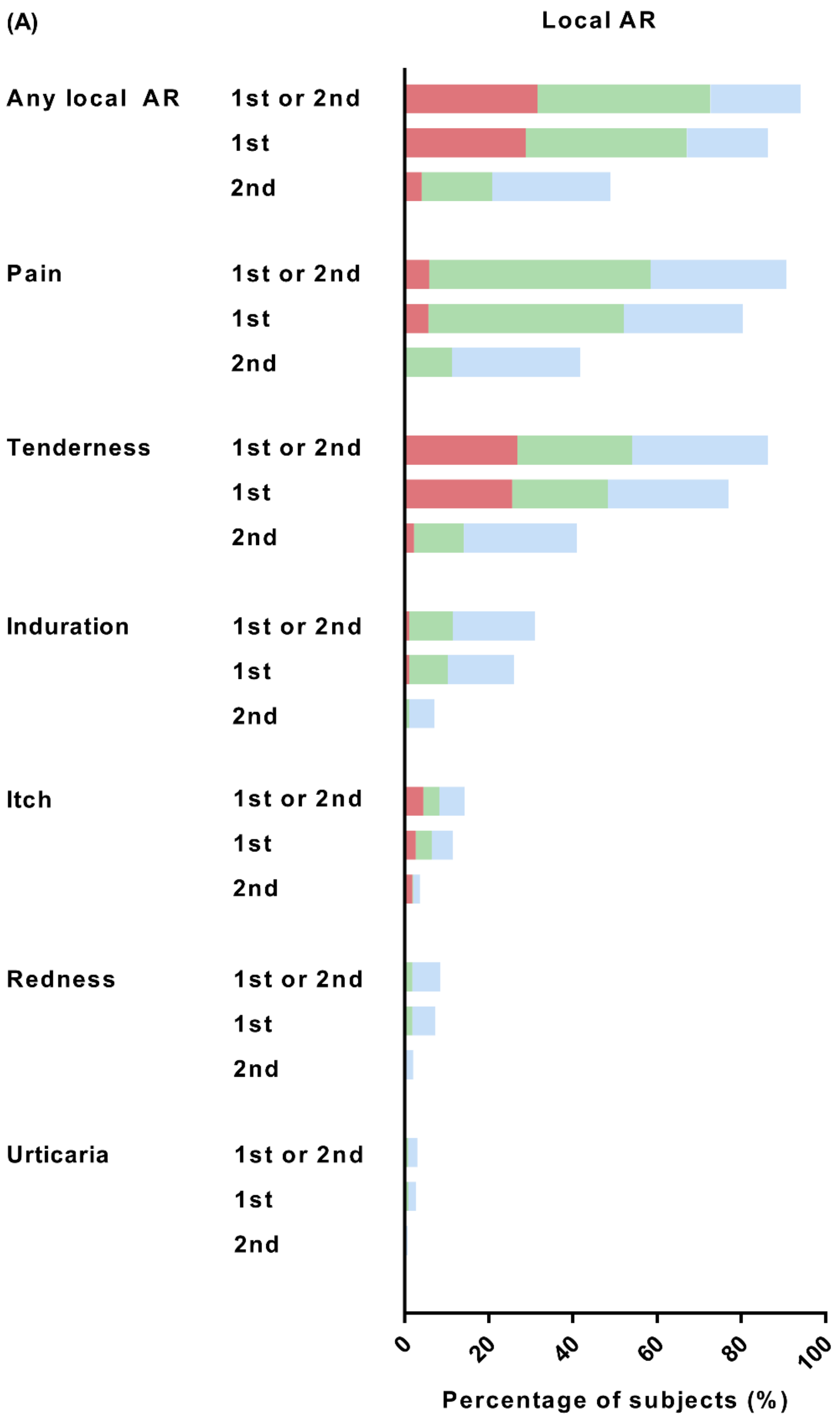

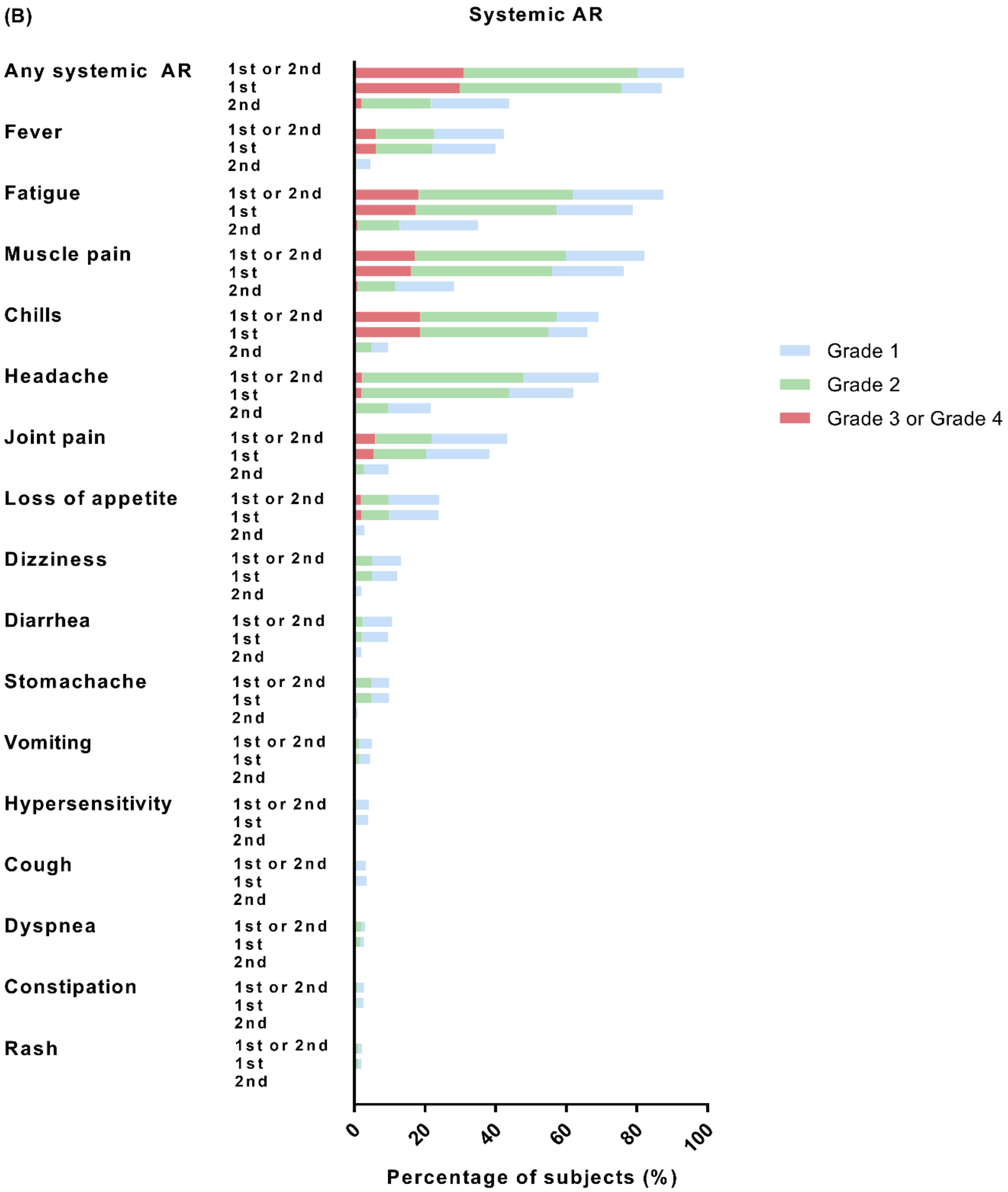

(B)
Systemic AR
Any systemic AR
Fever
Fatigue
Muscle pain
Chills
Headache
Joint pain
Loss of appetite
Dizziness
Diarrhea
Stomachache
Vomiting
Hypersensitivity
Cough
Dyspnea
Constipation
Rash
1st or 2nd
1st
2nd
0
20
40
60
80
100
Percentage of subjects (%)
Grade 1
Grade 2
Grade 3 or Grade 4
