# Supplementary material for: Anti-SARS-CoV-2 Spike Protein RBD Antibody Levels After Receiving a Second Dose of ChAdOx1 nCov-19 (AZD1222) Vaccine in Healthcare Workers: Lack of Association With Age, Sex, Obesity, and Adverse Reactions
Source: Front Immunol. 2021 Nov 25;12:779212. doi: 10.3389/fimmu.2021.779212 (PMC8654782; doi:10.3389/fimmu.2021.779212)
Supplement: Supplementary file 3 [file Image_3.pdf]

Supplementary Figure 3. Distribution of log-transformed (ln) antibody concentration at 4 weeks after the second injection of ChAdOx1 nCoV-19 vaccine according to severity of (A) any adverse reactions, (B) local adverse reactions, and (C) systemic adverse reactions after the second injection of ChAdOx1 nCoV-19 vaccine. Antibody was expressed as the natural logarithm of concentration of anti-SARS-CoV-2 S protein RBD. Upper and lower whiskers represent the highest and lowest data points, respectively, excluding any outliers. The horizontal lines in the middle, top, and bottom of the box represent the median, 75th percentile, and 25th percentile, respectively.

(A)

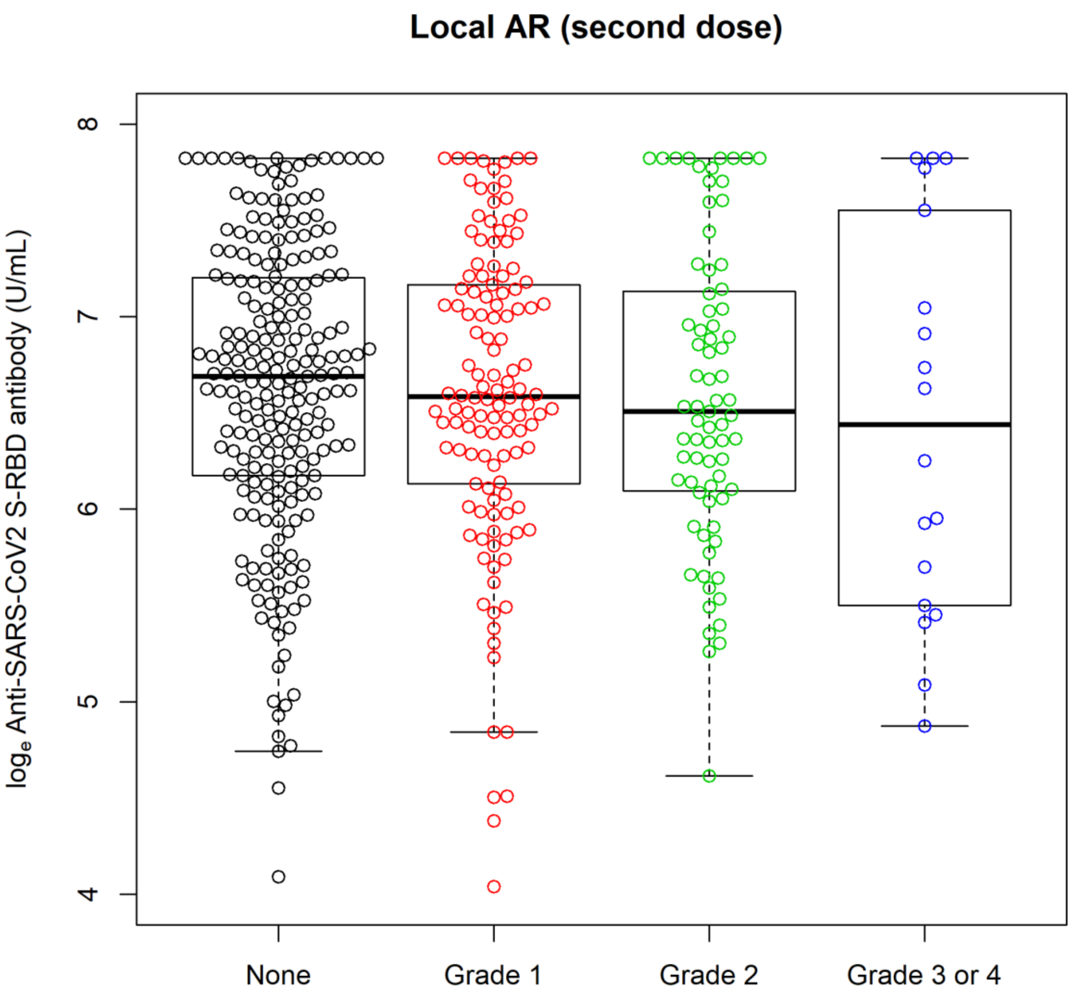

(B)

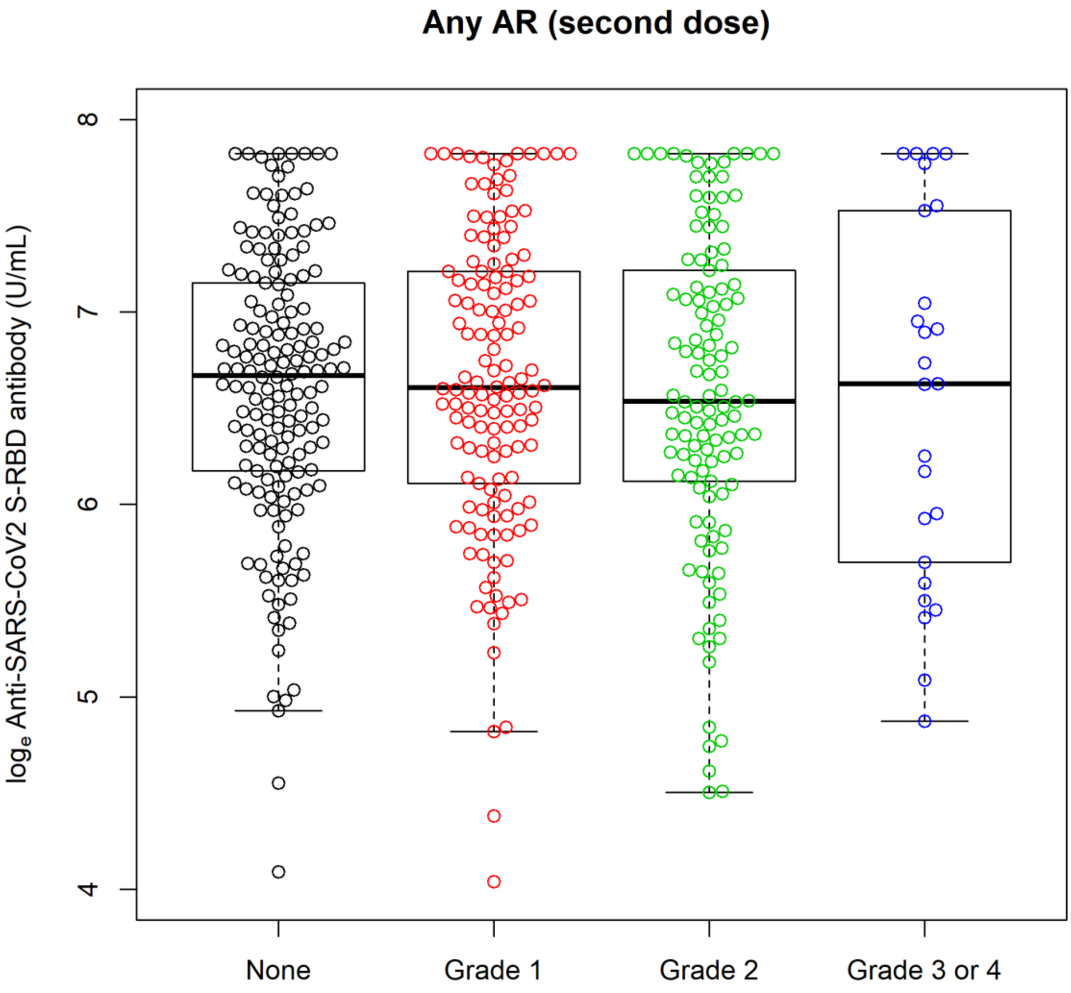
Any AR (second dose)
log$_e$ Anti-SARS-CoV2 S-RBD antibody (U/mL)
8
7
6
5
4
None
Grade 1
Grade 2
Grade 3 or 4

(C)

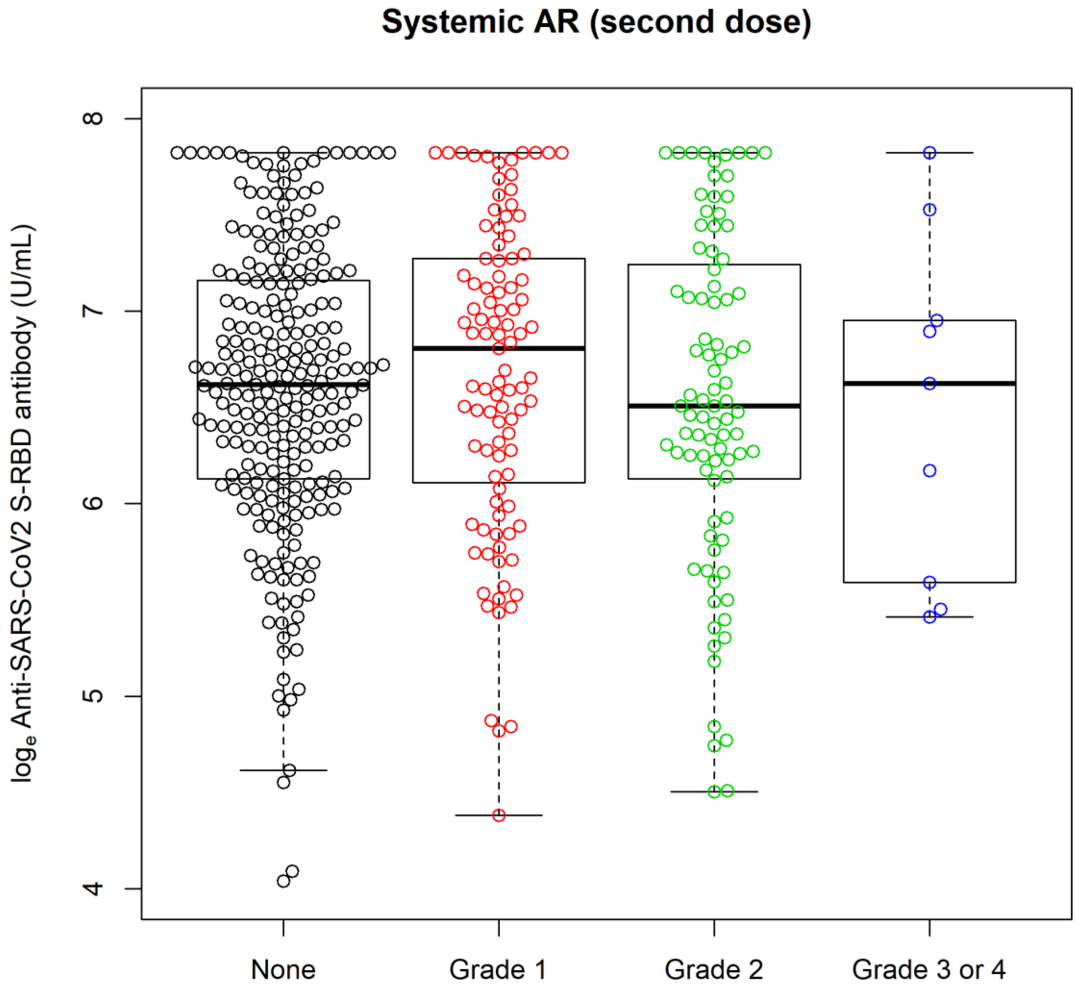

Systemic AR (second dose)
loge Anti-SARS-CoV2 S-RBD antibody (U/mL)
4
5
6
7
8
None
Grade 1
Grade 2
Grade 3 or 4
